# Supplementary material for: Intracellular Redox-Balance Involvement in Temozolomide Resistance-Related Molecular Mechanisms in Glioblastoma
Source: Cells. 2019 Oct 24;8(11):1315. doi: 10.3390/cells8111315 (PMC6912456; doi:10.3390/cells8111315)
Supplement: Supplementary file 1 [file cells-08-01315-s001.pptx]

## Slide 1
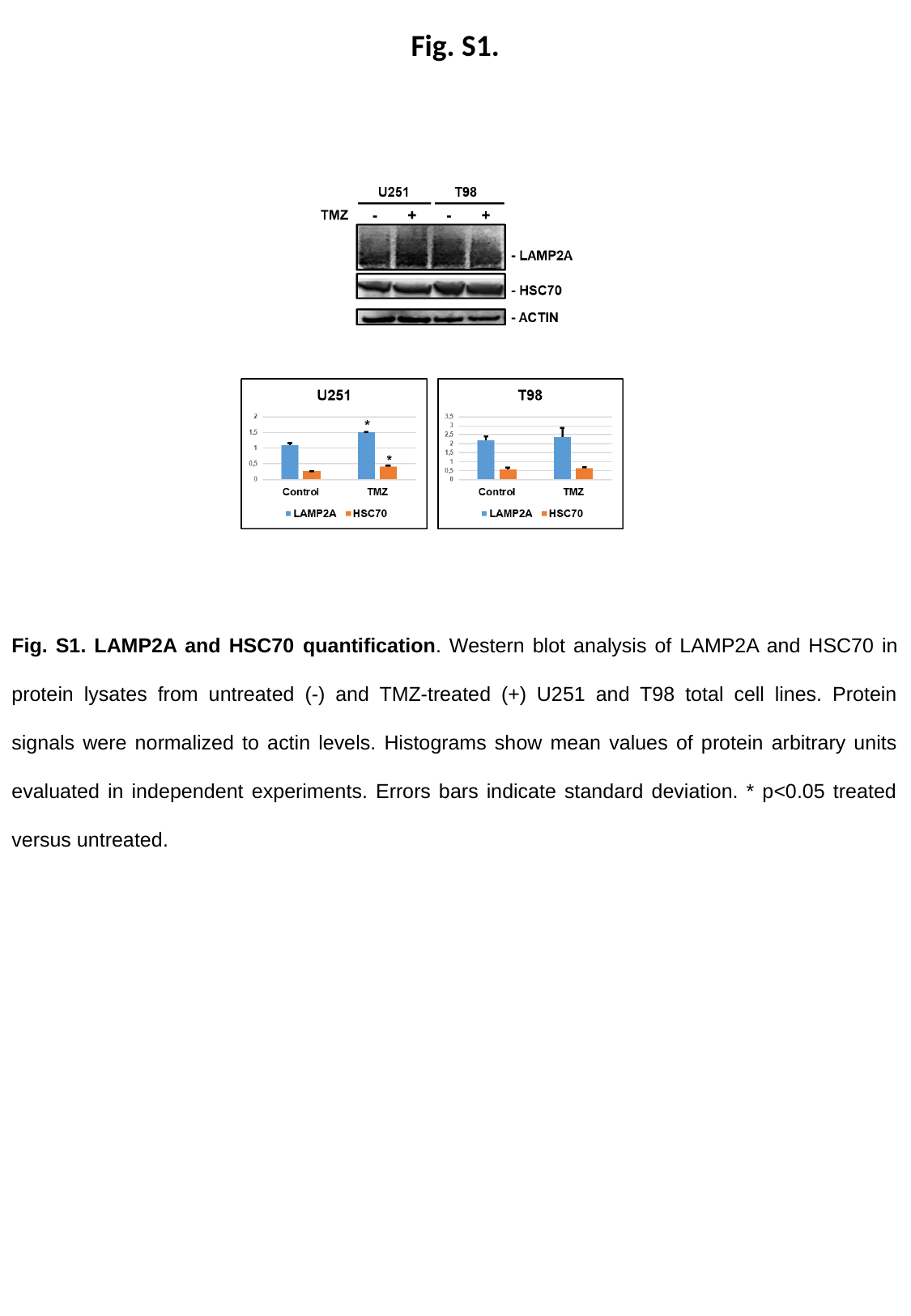

Fig. S1.
Fig. S1. LAMP2A and HSC70 quantification. Western blot analysis of LAMP2A and HSC70 in protein lysates from untreated (-) and TMZ-treated (+) U251 and T98 total cell lines. Protein signals were normalized to actin levels. Histograms show mean values of protein arbitrary units evaluated in independent experiments. Errors bars indicate standard deviation. * p<0.05 treated versus untreated.

## Slide 2
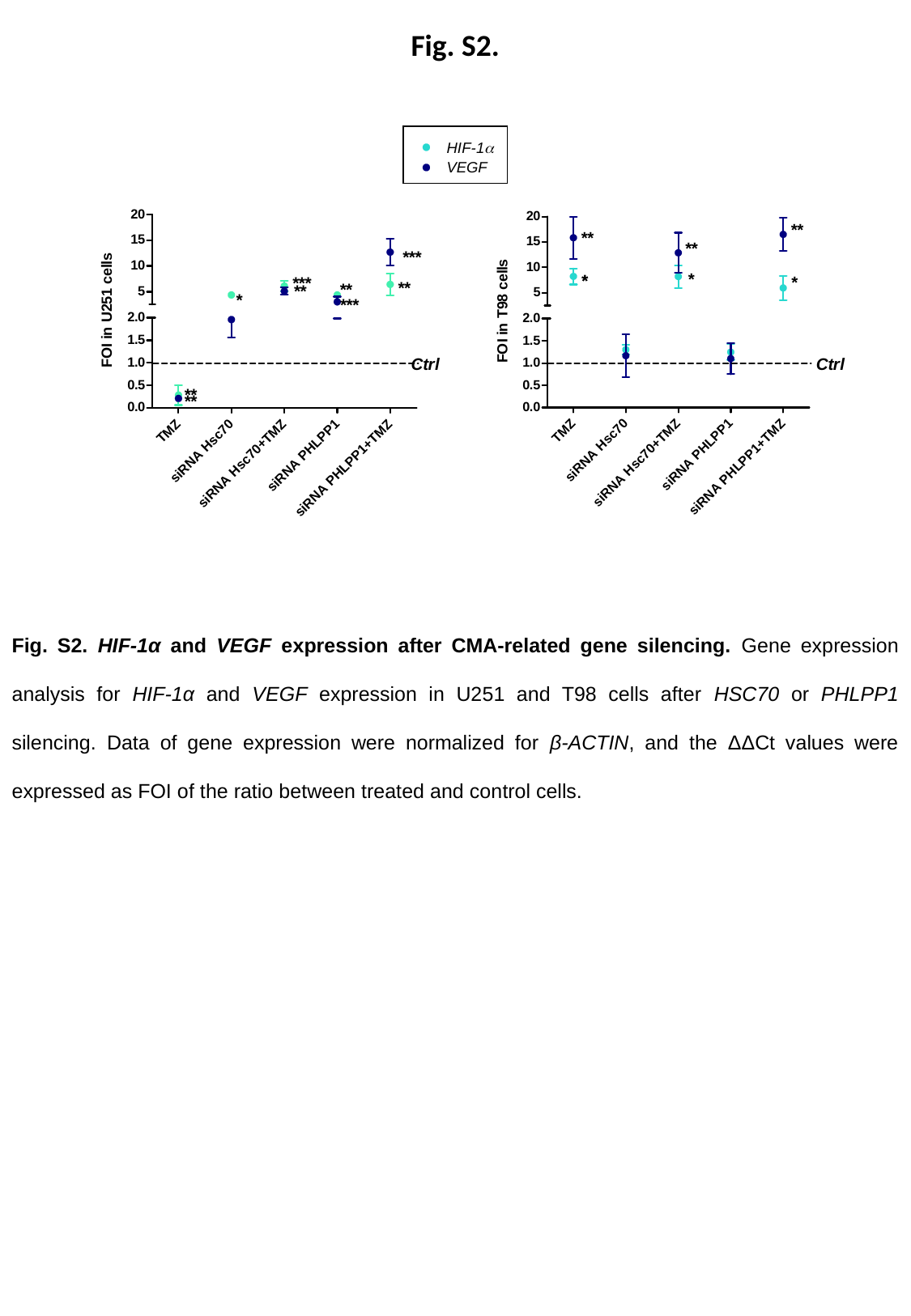

Fig. S2.
Fig. S2. HIF-1α and VEGF expression after CMA-related gene silencing. Gene expression analysis for HIF-1α and VEGF expression in U251 and T98 cells after HSC70 or PHLPP1 silencing. Data of gene expression were normalized for β-ACTIN, and the ΔΔCt values were expressed as FOI of the ratio between treated and control cells.

## Slide 3
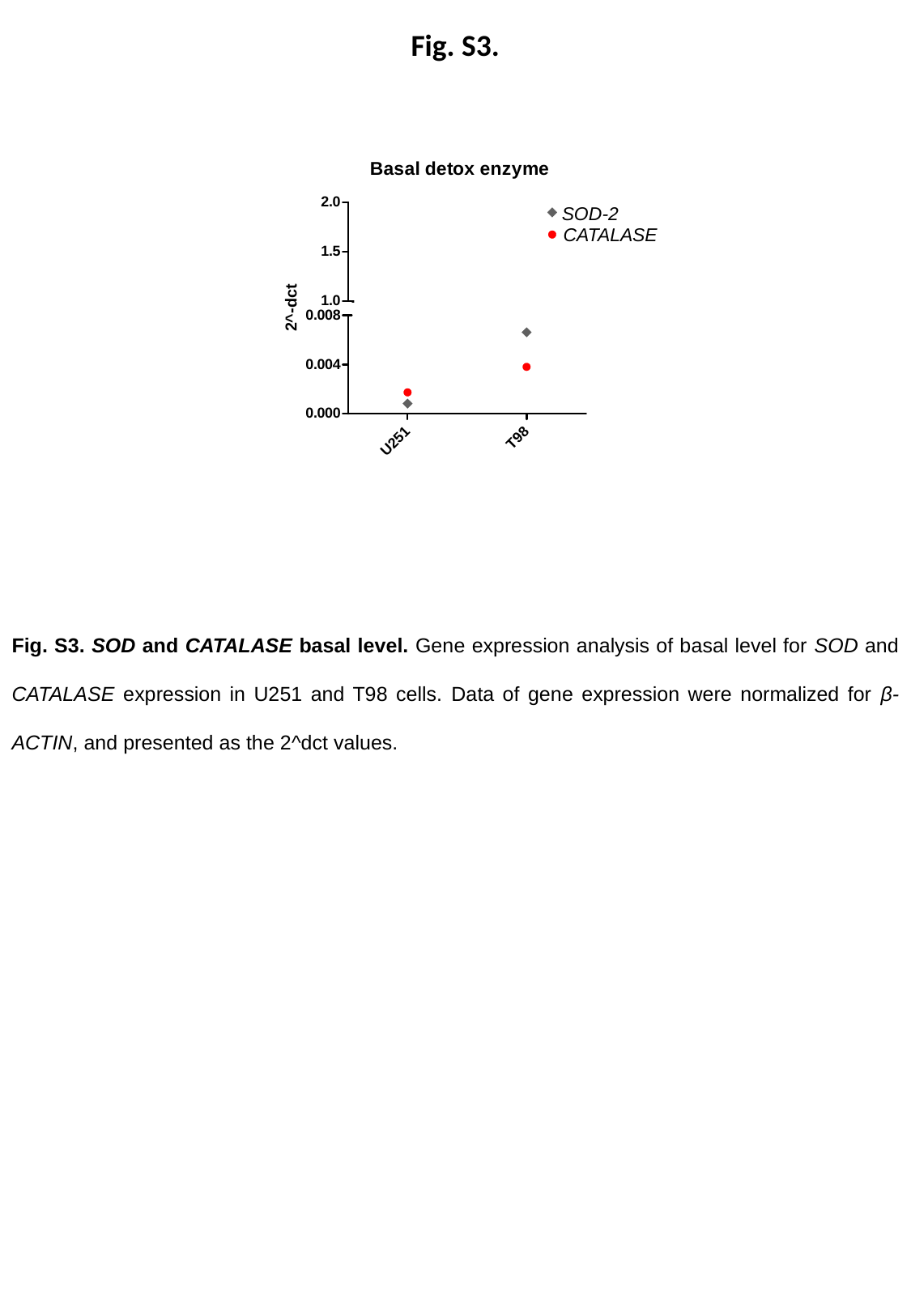

Fig. S3.
SOD-2
CATALASE
Fig. S3. SOD and CATALASE basal level. Gene expression analysis of basal level for SOD and CATALASE expression in U251 and T98 cells. Data of gene expression were normalized for β-ACTIN, and presented as the 2^dct values.
